# Books Received and Reviewed

**Published:** 1923-01

**Authors:** 


### BOOKS RECEIVED AND REVIEWED

Clinical Periodontia, by Paul R. Stillman, D. D. S. Instructor of Periodontia, Columbia University, New York, Past-President of the American Academy of Periodontology, and John Oppie McCall, A. B., D. D. S., instructor in Periodontia, Columbia University, New York, formerly professor of Oral Hygiene and Periodontia, Dental Department, University of Buffalo, New York; Past-President of the American Academy of Periodontology. This book represents the first serious attempt to deal with the subject of Periodontoclasia (so called Pyorrhea Alveolaris) in a really comprehensive way. The authors have each achieved a place among the foremost specialists in this field and to the knowledge gained through clinical success they have added a well rounded study of the Histology and Pathology of the investing tissues of the teeth together with the theories of etiology advanced by McCall and already widely accepted.

The studies of Stillman on Traumatic Occlusion are also given the fullest consideration and it is not too much to say that success in periodontic treatment can not be expected without a knowledge of this condition. This book is well printed, bound in maroon color cloth, contains 240 pages. The dissemination of knowledge which will assist in the control of Periodontoclasia furnishes the underlying motive for the publication of this excellent and comprehensive book, dedicated to the members of the American Academy of Periodontology, published by The MacMillan Company, New York. Copyrighted 1922, printed in United States. Clinical Periodontia is already among the best sellers which indicates its popularity. Price, $3.50.

Anthony's Dental Dictionary, by L. Pierce Anthony, D. D. S., associate editor of the *Dental Cosmos*, Chairman of the Committee of Nomenclature of the American

Dental Association. Published by Lea & Febiger, Philadelphia and New York. Price $4.50.

This Dental Dictionary, compiled by Dr. Anthony, is what should be termed the Unabridged Dental Dictionary, as it contains a complete list of all the older, well known, much used dental terms and besides all the new, later, synthisized words suggested by the most recent development in the dental practice. The book is set up in double column, has 324 pages and is bound in fabricoid leather—a very serviceable volume—is excellent for the general practitioner and indispensible for the dental speaker and thesis writer. There are also illustrations and color plates.

The preface states that the work is the result of the author's aim to produce an up-to-date dental dictionary and he has done it and done it well. The book is printed in the United States and copyrighted, 1922.

---

### NEW PUBLICATION BY C. V. MOSBY COMPANY

THRUST is a house organ to be published monthly by the C. V. Mosby Company. It will reflect the progress made from month to month by the Mosby Medical Journals, and to acquaint medical advertisers with this progress. It is one of the methods that the Mosby Company will employ to render a greater and better service. Without this objective no firm or individual engaged in commercial work will last.

Our earnest endeavor has been, and will continue to be, the rendering of the greatest service that we possibly can.

The managers and editors of this new magazine are Dr. L. A. Duck, Mr. Paul Knobe, and R. D. Waye, all of the C. V. Mosby personnel.

---

The following criticism and review of the book "Mondern Dentistry for the Laity." The classic of Dental Lore recently appeared in the *Journal of Oralogy.* Where it comments on the "treating of all abscesses" and that "it is good dentistry to save a dead tooth for its utility until the ultimate abscess comes," the argument goes into a jam for of course there is a diversity of opinion among the members of the dental profession as to the best policy. The way the writer understands the subject, the age of the patient is the determining factor. If young, abscess treatment is indicated, if old or middle age, extract. Even then the vitality of the patient is the deciding factor. This, of course, is also dependent upon whether the tooth is in good enough physical condition to save and if the adjacent gum tissue and process are in restorable and recouperable health. The "oncoming abscess" mentioned is a handy expression or seemed to be one in the explanation of this subject for an abscessed tooth treated in youth and saved very often abscesses in middle age or after or during a physical let down or sickness. So the expression "oncoming" may be qualified by "if vital forces of health are not retained." It is a big subject, very big, and takes a book to explain it. The *Journal of Oralogy* certainly hits on all twelve cylinders.

MODERN DENTISTRY FOR THE LAITY AND INDUSTRIAL DENTISTRY FOR THE CORPORATION, MODERN PREVENTIVE DENTISTRY AND INDUSTRIAL SAFETY WELFARE DENTISTRY. A classic of Dental Lore. By Alfred A. Crocker, Cincinnati, O. 1922. Fourth Edition. Published by the DENTAL REGISTER, the Magazine of Dentistry, Pioneer Dental Journal of the United States, Cincinnati, Ohio.

The attempt of Mr. Crocker to present to the public the importance of Dentistry to general health deserves commendation. In this little volume the object of which is "to develop an informed public" the effort is a worthy one for "we more readily accept things that we understand."

Industrial Dentistry, treated in a separate chapter, is fast becoming a leading question. An employer is more certain of the regular output of labor by keeping his employees in good health. Instead of permitting employees to get ill and lose their jobs through illness, their health is maintained and they are kept on the job. Mr. Crocker points out that to insure such efficiency, industrial plants should maintain dental clinics, in addition to the medical clinics, for general health is impossible without a healthy mouth. The recognition of the relationship between systemic diseases and oral diseases places dental service in our industries on a plane with medical service.

The chapter devoted to "Evidence of the Value of Preventive Dentistry to Industrial Corporations" consists of letters from leading industrial plants where dental clinics are established.

The chapter on "The Teeth" covers in a satisfactory manner the origin of tooth decay. Special emphasis is laid on the necessity of systematic examination of the teeth by the dentist. This is not meant to prevent decay but to prevent the extension of decay at the outset. The point is clearly explained but needs greater emphasis "that the dentist can properly examine the teeth where it is impossible for the individual to do it himself."

The prevention of pyorrhea is emphasized, although what pyorrhea is, is not made clear to the lay reader. The definition of the term is unscientific and somewhat confusing. Pyorrhea is defined as a "metabolic disturbance of the human system, which manifests itself by a salivary calculas deposit on the teeth, which in turn sets up gingival irritation of the gums, increasing the bacterial production of germs in the month to a pathogenic degree."

Prevention is unquestionably at all times preferable to cure. Writing on the effect of dead teeth retained i[illegible] the mouth, Mr. Crocker explains that when decay is extensive and reaches the nerve, it is removed "and by the present method of root-canal filling and treatment the tooth lasts very well for years." Mr. Crocker admits, however, that "in discussing decay, I mentioned the abscess which eventually comes at the roots of devitalized teeth." There is no need to fear "the oncoming of an abscess" for it can be detected by the dentist by the means of a radiogram. But what is the meaning of an oncoming abscess? He answers, "all we know about it is that it is Nature's way of getting rid of a dead tooth."

It would logically follow that the layman would agree with Mr. Crocker that "dead teeth" should not be retained in the mouth against Nature's will. "A good tooth in the jaw is worth ten on a rubber plate, but on the other hand, a set of false teeth is better than one tooth which is a focus of infection." The danger of the presence of a "dead tooth" in the mouth which is a "focus of infection" and which will "eventually develop into an abscess" is sufficient reason for the layman to form a habit of mouth care. Mr. Crocker, however, does not reach such a conclusion, much to our surprise, for he believes that the placing of the mouth in good condition includes on "treating of all abscesses" and that "it is good dentistry to save the dead tooth for its utility (mastication probably) as long as possible, for as long as it (the dead tooth) is in healthful [?] condition, it is far better than a false ventive dentistry than an insignificant, unnoticed focus of infection.

While there is a great need for the extension of lay education on mouth hygiene and prophylaxis, nothing short of a logically consistent publication with a clear vision will meet the demand. The question of dead teeth must be presented in a scientific manner and in accordance with all the facts at hand.

Mr. Crocker deserves credit for attempting to present the dental situation from the layman's point of view. In the main it is an interesting book of contemporary dental opinion.—*Journal of Oralogy.*

